# Sustainability within interventional radiology: opportunities and hurdles

**DOI:** 10.1186/s42155-023-00362-1

**Published:** 2023-03-20

**Authors:** Anouk de Reeder, Pim Hendriks, Helena Plug - van der Plas, Dirk Zweers, Philine S. M. van Overbeeke, Joost Gravendeel, Johan W. H. Kruimer, Rutger W. van der Meer, Mark C. Burgmans

**Affiliations:** 1 Department of Radiology, Leiden University Medical Center, Leiden, The Netherlands; 2 Department of Business-Society Management, Rotterdam School of Management, Erasmus University Rotterdam, Rotterdam, The Netherlands; 3 Martini Hospital, Groningen, The Netherlands; 4 Department of Radiology, Flevoziekenhuis, Almere, The Netherlands

**Keywords:** Interventional radiology, Healthcare sustainability, Sustainability in interventional radiology, Recycling, Waste, Water pollution

## Abstract

**Background:**

Healthcare is a highly polluting industry and attention to the need for making this sector more sustainable is growing. The interventional radiology (IR) department is a relatively unique department in the hospital because of its synergetic use of both imaging equipment and medical instruments. As a result, the interventional radiology department causes a significant environmental burden in terms of energy usage, waste and water pollution. The aim of this study was to explore the current state of sustainability within IR by conducting a survey and interviews among IR specialists in the Netherlands.

**Results:**

The main findings of this study were that there is a high awareness for the need of sustainability within IR, but that there is still limited action. Previous studies point towards the various opportunities in the field of energy, waste and water pollution, yet our study unveils these opportunities are often not implemented because of (1) sustainability not being a priority, (2) a dependency on employees, and (3) factors that simply cannot be changed by an individual IR department or hospital. Generally, our study indicates that there is a willingness to become more sustainable, but that the current system involves a wide range barriers that hinder true change. Furthermore, it seems that no one is currently taking the lead and a leading role from higher management, government, healthcare authorities or professional societies is lacking.

**Conclusions:**

Despite the hurdles found in our study, IR departments can implement several improvements. An important factor is that sustainability should not lead to lower convenience for employees, which can be ensured by a sufficiently designed waste infrastructure and behavioral nudges. Furthermore, there lies an opportunity in more collaboration between IR departments in knowledge sharing and open innovation.

**Supplementary Information:**

The online version contains supplementary material available at 10.1186/s42155-023-00362-1.

## Background

### Introduction

There is a scientific consensus of almost 100% on the fact that humankind causes global warming and the negative consequences of this are increasingly being recognized. Awareness among organizations and companies has grown over recent years and many now focus on sustainable development and limiting their environmental impacts (Myers et al. 2021; Steffen et al. 2015). Healthcare has a significant environmental burden and accounts for about 7% of the total CO_2_ footprint in the Netherlands. However, healthcare has stayed behind in terms of sustainable development compared to other sectors (Rodriguez et al. 2020). Hospitals are significant polluters and account for about 35% of total emissions of health services (Keller et al. 2021).

The interventional radiology department is a relatively unique department in the hospital because of its synergetic use of both imaging equipment as well as medical instruments and iodinated contrast media needed for interventions. As a result, the interventional radiology department causes a significant environmental burden in terms of energy usage, waste, and water pollution (Chawla et al. 2017). This entails that making IR more sustainable requires actions in all of these fields. A handful of studies on sustainability in IR have been published, presenting insights on the environmental impact of an IR department and actions that IR specialists can take themselves (Brassil & Torreggiani 2019; Chua et al. 2021; Shum et al. 2022).

The highest greenhouse gas emissions in an IR department are caused by the Heating, ventilation, and air conditioning (HVAC) system and half of the emissions of the HVAC systems are generated during out of office hours (Chua et al. 2021). Similarly, two-thirds of energy usage in CTs is due to non-productive (idle) states (Heye et al. 2020). Energy usage can be reduced significantly by using ‘energy saving’ or stand-by modus, occupancy sensors, and turning off the Picture Archiving and Communication System monitors when not in use. (Thiel et al. 2018; Chawla et al. 2017; Prasanna et al. 2011).

The second highest source of greenhouse gas emissions in the IR department is the use of single-use medical instruments due to production and delivery (Chua et al. 2021). Furthermore, single-use instruments cause high amounts of waste, not only by the instruments itself but also by their packaging. On average waste packaging accounts for over half of the total weight of the single-use medical product (Clements et al. (2020). Most of this waste is potentially recyclable. However, the success of recycling depends on recycling awareness and the behavior of employees. (Clements et al. 2020).

Another important environmental burden is water pollution caused by iodized contrast media (Dekker et al. 2022). Once in the water, these contrast media break down badly, allowing them to accumulate. Interventions to reduce water pollution by contrast media are relatively easy and do not need substantial investments (Dekker et al. 2022; Hoogenboom et al. 2021).

Despite this knowledge, the current state of sustainability in IR departments in practice is unclear in terms of awareness of the subject, actions undertaken and responsibilities. The aim of the study was to analyze the current state of sustainability in IR departments in the Netherlands with respect to awareness, actions undertaken and organization with the ultimate goal of identifying opportunities, barriers and guidance in making IR more sustainable.

## Methods

To identify these opportunities, barriers and guidance in making IR more sustainable, a mixed methods approach was used consisting of a survey and semi-structured interviews. The online survey was set up in Qualtrics and distributed per mail via the Dutch Society of interventional radiology (Dutch: Nederlandse Vereniging voor InterventieRadiologie, NVIR). No approval of an ethical board was needed for this survey study amongst colleagues. Participants were informed that the study was about sustainability in interventional radiology and they would participate on a voluntary basis. Confidentiality and anonymity were ensured. In the survey, participants were asked to indicate to what extent they agreed (7-point Likert scale, 1 = Strongly disagree; 7 = Strongly agree and no opinion option) with the six following statements:

1. As IR department, we are aware of our negative impact on the environment (e.g. waste production, energy consumption).
2. As IR department, we have quantified our impact on the environment, for example with a life cycle analysis (LCA).
3. As IR department, we have taken actions to reduce our negative impact on the environment.
4. We have set up a green team within the IR department to make the department more sustainable.
5. If employees within the IR department themselves have an idea within the context of sustainability, then they know where to go with this idea to develop it further.
6. In the context of making our IR department more sustainable, we work together with other departments in the hospital, for example by knowledge sharing.

Excel (Microsoft Corporation 2018) and the statistical software package SPSS version 27 (IBM Corporation 2020) were used for data analytics using descriptive statistics and visualization. No additional statistical analyses were performed.

Additional semi-structured interviews were conducted to allow for examining underlying dynamics and in-depth information. The sample of interviewees (Table 1) was gathered through convenience sampling in the IR department of the LUMC and by making use of the survey. At the end of the survey, respondents were given the option to leave their contact details if they were willing to participate in a follow-up interview about the topic. Three interventional radiologists, a chief IR technician responsible for purchase of equipment and materials, an IR technologist and a (non-IR) medical technology employee with extensive knowledge about medical equipment and legislation were interviewed following a semi-structured interview protocol (see Appendix).

**Table 1:** Overview of interviewees

Interviewee code	Staff function
I1	IR technologist
I2	Medical technology employee
I3	Section head IR
I4	Interventional Radiologist (responsible for procurement)
I5	Interventional radiologist
I6	Interventional radiologist

The semi-structured interviews each lasted between 24 and 37 min and were recorded and transcribed, resulting in 28 pages of raw data. The interviews were then analyzed using thematic analysis methods which allowed for the identification and analysis of themes (patterns) within the data (Braun & Clarke 2006).

## Results

**Fig. 1:**
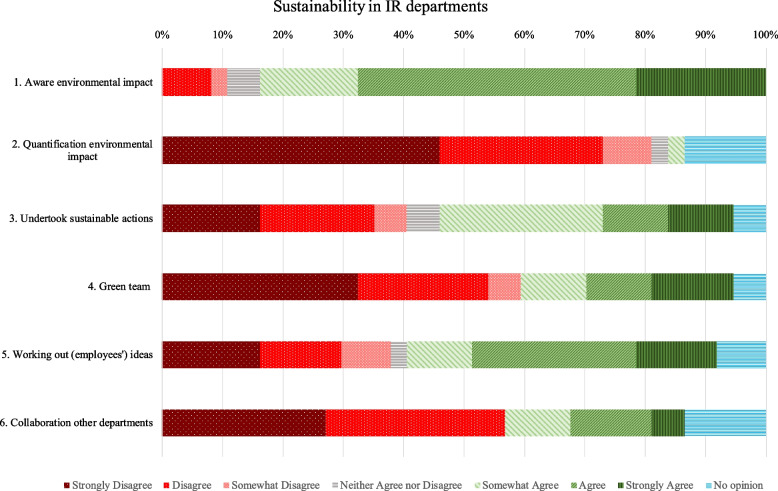
Results of online survey

The survey was distributed through e-mail to all 272 NVIR members. In total, 58 people opened the survey, which resulted in a response rate of 21,32%. Out of these 58 people, 37 people completed the survey, which resulted in a completion rate of 63,79% and a total response rate of 13,6%. Figure 1 displays the results of the online survey.

The survey shows that a large majority (83.7%) of the respondents indicated that they are aware of the negative environmental impact of their IR service. However, 30% of the respondents that indicated awareness of their environmental impact had not taken sustainable actions (Q3). This group was asked the follow-up question as to why this could be the case. The reasons mentioned are ‘the structure of the organization’, ‘not knowing where to start’, ‘no alternatives available for disposables’, ‘lack of time’ and ‘impeding rules’. 40,5% of all respondents indicated that their department had taken action (Q3). Actions mentioned were in the field of energy and waste, like as separating waste and turning off lights/equipment when not needed. Only one respondent mentioned an action in the field of water pollution.

Almost no IR department (87,2%) quantified their environmental impact (Q2). In terms of organization, green teams seemed to be quite common as more than one third of the respondents indicated that they had set up a green team to make their department more sustainable. About 70% indicated that they did not (yet) collaborate with other departments in the hospital in the context of sustainability, or that they had no opinion about this.

### Results: interviews

**Fig. 2:**
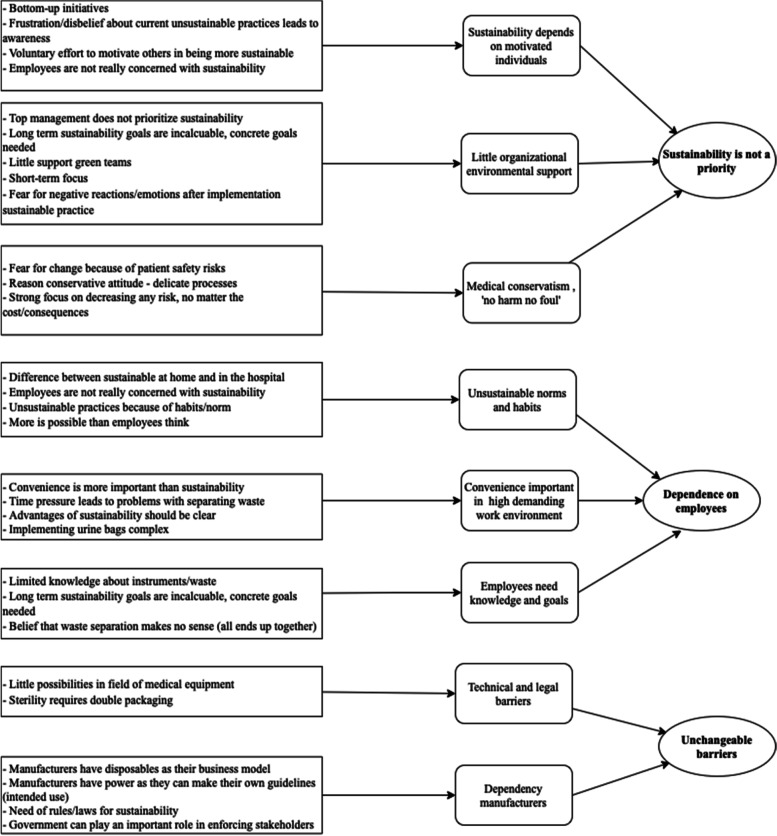
Coding tree (interviews)

Four internal IR employees were interviewed. Furthermore, in total five respondents left their contact details for a follow-up semi-structured interview in the survey. This resulted in two additional semi-structured interviews (response rate = 40%). Figure 2 displays the coding tree with the first-order codes, second-order themes, and aggregate dimensions. The three aggregate dimensions were labeled as follows: ‘sustainability is not a priority’, (1), ‘dependence on employees’ (2) and ‘unchangeable barriers’ (3).

#### Sustainability is not a priority

The first aggregate dimension is about the general notion that sustainability is merely seen as a ‘side’ project and depends on motivated individuals. Sustainability is currently mostly a bottom-up activity that relies on intrinsically motivated individuals. Generally, it seemed that awareness of sustainability is growing, but that it was not considered a core element of the organization (I1, I4, I7). Motivation for sustainability was often triggered by frustration or disbelief about current unsustainable practices, like seeing the amount of waste that is produced every day and by noticing differences between behavior at home and in the hospital. Besides, motivation came from a general awareness that humans use too many resources and things must change (I1).

Multiple interviewees mentioned that top management should take a more proactive approach, as currently “everyone is just doing something” (I2). Furthermore, employees experienced little support by top management in setting up a green team: *“*They started years ago with that and it wasn’t supported at all by higher management. There are also other things that need your attention, that made it water down*”* (I1).

Finally, patient safety concerns affected sustainability in the way that environmental burdens are taken for granted as long as patient safety is maximized. This seemed to arise from the ‘no harm, no foul’ attitude in healthcare. There were numerous practices and guidelines that are meant to lead to more safety without taking the environmental burden into consideration, while there is often mixed or limited evidence that supports these practices. An example can be found in the use of disposable gloves, which leads to high amounts of waste. It is not clear whether gloves should be changed after each contact with a patient, whether they can be used for longer periods of time, or whether washing hands thoroughly is equally safe (I2). Yet changing this can lead to fear, partly caused by the fact that the use has been so ingrained for many years (I2):

“Still, that [disposable gloves] gives a kind of false safety, because you have the idea that you are well protected by it, but if we read the rules carefully we don’t always have to wear those gloves.” (I2)

Another example of this specifically found in IR lied in shutting down machines at night. Procedures regarding shutting down equipment differed per hospital and were often surrounded by lack of clarity in the field of responsibility and monitoring whether the equipment is actually shut down. Reasons to not put off systems were the start-up time and potential technical problems in case of an emergency in which the angiography system or CT-scanner is immediately needed (I1). Yet, this was based on a ‘gut feeling’/common sense and not on a thorough risk analysis in which the frequency and severity of these instances is researched (I3). Making proper considerations about certain practices and their risks requires research in the form of risk-analyses. However, currently there is no department or team that is responsible for doing these kinds of analyses.

#### Dependence on employees

The second aggregate dimension describes the dependence on employees, in the way that employees are crucial in the success of becoming more sustainable, but that there are multiple factors that play a role in this.

All interviewees mentioned a general perception that most colleagues seem to not be too concerned with sustainability. Several interviewees (I1, I4) mentioned a difference between sustainable behavior at home and at the hospital and that people seem to care less at their workplace: *“*I sometimes say rather cynically that if people could take the energy to save home with them, they would do it*.”* (I1). This was mostly in ‘smaller’ sustainability practices like putting off lights and separating waste properly. In principle, computers and lights are to be put off when the working day is over, but in practice they are often still on (I3, I4, I1). Yet, even the employees that were in fact concerned with sustainability mentioned that they did not always act if they think something could be done more sustainable, simply because it is ‘just the way things go here’:

“I don't think they [colleagues] care that much about it. Neither do I when I'm at work. You're in a certain flow and with certain things, but I'm not going to call the pharmaceutical company to ask if they could put a layer [packaging material] less around it.” (I4)

Furthermore, employees often perceived that little change is possible because current practices arise from patient safety regulations and protocols, whereas actually these practices are habits and not formally established. This was found in the previously mentioned example of shutting down angiography systems and CT-scanners at night. When asked whether (a portion of) equipment could be shut down if no one was there (e.g. at night), I1 believed it could not be changed. Yet, employees from the medical technology department indicated that there was no official regulation to leave devices on; it was merely done out of habit and in a ‘better safe than sorry’ perspective.

Besides, employees may lack relevant knowledge to execute sustainable behavior. Sustainability-related targets were hospital-wide and mainly relevant for the facility department as they focus on ‘large’ goals like energy and waste total (I6). There was a lack of specific sustainability goals for departments, which decreased the ‘need’ to execute sustainable behavior as employees had no idea what they were actually contributing. An example of a lack of knowledge to execute sustainable behavior properly lied in the field of materials and waste. Employees were expected to segregate different types of waste, like paper and plastics. Yet, employees often lacked proper knowledge about what waste belongs in which bin, for example with different types of plastics (I1). Employees also did not receive any formal training about this (I1, I4). Furthermore, the benefits of sorting waste were not clear, and among some employees there was a persistent belief that it does not make sense to sort waste, as they believe it all ends up on the same heap anyway (I1, I3).

Lastly, hospital workers are generally very busy and time pressure is often high, so working procedures are as efficient as possible and people are generally very hesitant to changes that lead to lower efficiency and convenience (I4, I5, I3). People often perceived that working more sustainably leads to lower convenience, which in turn leads to hesitance and experiencing it as a hassle: “we [doctors] are always irritated when there are too few things on the table, but that does imply that there is always too much and that we have to throw things away.” (I4); “A lot of people also think it [separating packaging waste] is a hassle, which is also partly true because we want them to hand over things quickly, and then they have to unpack it quickly and then throw it away somewhere else” (I4). Furthermore, employees already had a lot of administrative tasks and lots of rules and procedures they had to comply with. Adding more (sustainable) tasks made them feel overwhelmed:

“People already have so much on their minds and then additional rules are added, they already feel they have so much to do, write down and register. Then they have six bins in front of them and then they also have to think about where to put it.” (I3)

Employees do not like lower convenience, yet they might be more willing to accept changes if the advantages or reasons for sustainable products are clear, which currently often does not seem to be the case (I2).

#### Unchangeable barriers

The last aggregate dimension is about barriers that are not possible to overcome as an individual IR department. Unchangeable barriers can be found in two forms: legal and technical barriers (I3, I5). There are certain rules that ensure safety, but hinder sustainability. A large part of hospital waste comes from packaging, but sterile products must be double-wrapped according to the law (I3). Sustainability can also be hindered by technical barriers. Some types of equipment, like MRI-scanners, simply cannot be put off because they need permanent cooling (I3, I1). Besides, especially older equipment is not designed to be shut down and put on regularly, which has led to complicated starting procedures. At the time they were designed, this factor was simply not taken into consideration.

Furthermore, in terms of making materials and equipment more sustainable, hospitals depend on manufacturers, which do not always seem to be a frontrunner in this field. A substantial part of the environmental burden of healthcare is caused by the use of single-use instruments, which were pushed into the market by manufacturers selling them as cheaper, safer, and more convenient (I4). Yet, single-use instruments cause a lot of waste, not only from the instrument itself, but also by its packaging. However, it is not easy to switch to reusables, simply because for many instruments manufacturers do not produce a reusable option (I5). The business model of manufacturers is built around single-use materials, which makes them hesitant in changing their business models as single-use materials are more profitable (I2). Furthermore, manufacturers possess power in the way they can compose the ‘intended use’ by themselves, which describes how the healthcare professional should use the instrument (I3). Because of this, medical professionals are obliged to throw away a single-use material, even if it is technically possible to sterilize and reuse it. Not following the intended use can (theoretically) be done, but the consequence is that the responsibility if something goes wrong moves to the medical professional or the hospital, and there could be legal consequences. The government could play an important role in enforcing rules that force manufacturers to change their business model, but currently the government seems rather passive (I2).

## Discussion

Previous studies show that there are opportunities in the field of energy, wastage and water pollution. Yet, these opportunities are often not implemented. Our study shows that awareness for the need of sustainability among IR staff is high, but that it often does not lead to action due to a variety of reasons, such as sustainability not being a priority, the dependency on employees, and factors that simply cannot be changed by an individual IR department or hospital. Despite these hurdles, our study also unveils several possibilities and actions that IR departments can take to become more sustainable.

Firstly, both the survey and interviews indicated that whereas most IR departments are taking (some) actions, they lack quantification on their own environmental impact. This hinders setting goals, identifying priorities, and measuring progress (Keller et al. 2021). Furthermore, it appears that actions undertaken do not focus on the core activities and procedures of IR. Instead, actions are rather obvious and similar to actions that people can take at home, like separating waste and turning off lights when leaving. This is in line with previous studies, as there has been little focus on implementing sustainability in key activities and procedures of IR (Brassil & Torreggiani 2019). Implementing sustainability in the core activities of healthcare is further complicated by a lack of sustainability standards and assessment methods in healthcare, like the Building Research Establishment Environmental Assessment Method (BREEAM) in the building sector (Shan & Hwang 2018).

Furthermore, it stands out that water pollution caused by iodized contrast media is a topic that already has gained attention in research, yet in practice it seems to be an unknown and overlooked topic among IR staff. However, previous research presents readily available actions that IR departments can undertake (Hoogenboom et al. 2021). A relatively easy and cheap intervention to avoid water pollution lies in collecting urine with contrast agents with urine bags, as shown in a recent trial study in six Dutch hospitals (Hoogenboom et al. 2021). In urine bags, the urine is converted into a gel-like substance which can be thrown away with residual waste. The trial showed that the willingness among both staff and patients is high and that it does not cost much extra time and money to hand out the urine bags, and thus that it provides for an easy and low-cost way to avoid water pollution (Hoogenboom et al. 2021). Another method to avoid water pollution involves reducing the dose by basing it on the patient's weight and the procedure. In addition, a multi-patient system can avoid wastage, by allowing the use of different vial sizes, and by reusing leftover contrast media (Dekker et al. 2022; Hoogenboom et al. 2021). A third method involves collecting unused contrast medium in a special box, after which it ends up in a waste stream that will be incarnated. This method is currently being developed in a program on recycling unused iodine by GE Healthcare (Dekker et al. 2022).

A crucial factor in the success of sustainability interventions is the behavior of employees. Interviews pointed out that there are various factors that (negatively) affect this behavior. As healthcare employees are already busy and have lots of tasks, being more sustainable should not be experienced as a hassle. For proper waste sorting/recycling behavior, two factors are essential: simplicity in the form of a sufficiently designed recycling infrastructure and adequate knowledge regarding recycling (Johansson 2016). The first factor involves the design of the waste bins, but also the physical distance to a recycling bin. A study by Brassil and Torreggiani (2019) about improving sustainability at the IR department also touches upon this. They concluded that the position of the clinical waste bin, in which waste is collected that can pose risks of infection, is critical in appropriate segregation of waste. Placing this bin near the scrub station led to the disposal of non-risk paper towels and thus waste in the wrong waste stream. Furthermore, they also found that if the clinical waste bin was closer to the interventional radiologist than recycling bins during procedures led to a difference of up to 60% of wrongly segregated waste. They found that placing the recycling bin closer to the interventionalist from the beginning of procedures and keeping the clinical waste bin further away led to instant improvement and behavioral changes.

Specific knowledge regarding recycling, the second essential factor for recycling behavior, involves on the one hand knowledge about the benefits and importance of recycling and on the other hand practical knowledge about where and how to recycle. This also involves communication of what waste belongs in which bin, which can be influenced by the design of the bin and the use of labels. Another way to improve sustainable behavior could lie in 'nudging'. Nudging is a way of influencing behavior in a positive way and without forbidding ('bad') things, especially through design and logistic choices (Thaler & Sunstein 2008).

An additional challenge with regards to sustainability specifically found in healthcare that emerged from our research lies in possible tensions with patient safety. As sustainability is currently not treated as a top priority, whereas patient safety is, a ‘better be safe than sorry’ approach is followed. To take both patient safety risks and environmental consequences into consideration, risk-analyses are essential. Furthermore, a critical look might have to be taken at whether current safety practices are based on the current state of science and thus actually lead to higher safety (Hansson 2020) and whether practices are done out of habit or are actually obligatory.

In terms of organization, green teams seem to be a rather popular method to execute sustainable change. Yet, IR departments should not consider setting up a green team as a quick win, as keeping such a team running is challenging. This is because it seems to be a voluntary task on top of daily job activities.

Both the survey and interviews pointed out that there lies an opportunity in more collaboration between (1) other hospital departments and (2) IR departments in the Netherlands in the field of sustainability in sharing knowledge and so to ‘not reinvent the wheel’. Sharing knowledge between different organizations is called “open innovation” and can lead to significant improvements in sustainability (Lopes et al. 2017). A common knowledge-sharing strategy in the healthcare sector is knowledge brokering (Waring et al. 2013). A knowledge broker forms interdepartmental relationships to facilitate the creation, use and sharing of knowledge. In the context of the hospital, it has been found that employees with a 'hybrid' organizational role (e.g., a doctor with management tasks) are best suited for this. Knowledge brokering could be applied both internally (between different departments of the hospital) and externally (between different interventional radiology departments). Health authorities and professional medical societies (like the NVIR in the Netherlands) could play a role in this, as in principle they already provide an 'infrastructure' for cooperation. On top of providing benefits related to knowledge sharing, collaboration could also provide advantages in terms of more radical changes to ‘truly’ become sustainable. Implementing drastic changes in terms of materials and waste, like switching from single-use to reusable instruments, depends on manufacturers. Acting as one group/body might give a stronger negotiating position against these manufacturers and put more pressure on them to change.

Lastly, our study shows that sustainability is currently mostly a bottom-up, intrinsic activity that relies on motivated individuals. Employees generally experience little support from higher management. Higher management could play an important role in prioritizing sustainability in order to truly embed it into the organization. Moreover, the role of the government and healthcare authorities should not be overlooked, as they can impose rules and standards. The Dutch government already has taken some action, as indicated by the involvement in the ‘Green Deal Sustainable healthcare’ in which agreements are made (Green Deal Duurzame Zorg | RIVM, n.d.). However, there do not seem to be clear deliverables and involved organizations are not held accountable, which shows resemblance to our findings in the way that that sustainability in healthcare is currently voluntary and relies on taking personal initiative. Thus, a more proactive approach from the government is needed for more radical changes (Sherman et al. 2020).

A limitation of this study is external validity due to the focus on IR departments in the Netherlands only. Findings might be less applicable in other countries because of cultural differences. Furthermore, internal validity might be violated by the indications of sampling bias (due to the low response rate) and potential social desirability bias due to the self-report nature of the survey. Yet, our research design included several factors that counteract social desirability bias, like ensuring anonymity and confidentiality and using neutral wording (Vesely & Klöckner 2020). Furthermore, this bias was counteracted by triangulation made possible by the mixed method approach, which increases internal validity (Modell 2005).

A suggestion for future research in the field of sustainability within IR lies in making procedures itself more sustainable; in other words: implementing sustainability in the core activities of IR, instead of incremental changes. Furthermore, the potential of prevention should not be overlooked, as “prevention is better than cure” and a cure not given is always more sustainable (Seifert & Guenther 2019; Sherman et al. 2020). Lastly, future research could focus on how IR healthcare societies like the Cardiovascular and Interventional Radiological Society of Europe (CIRSE), Society of Interventional Radiology (SIR) and national societies like NVIR could play a role in making IR more sustainable, for example by organizating joined initiatives and establishing networks.

## Conclusion

Healthcare is a highly polluting industry and attention to the need for making this sector more sustainable is growing. Interventional radiology (IR) causes a significant environmental burden in terms of energy usage, waste and water pollution. Research on making IR more sustainable is limited and mainly focuses on specific actions that can be taken. To our knowledge, this was the first study to look at the current state of sustainability within the field of IR using a mixed-method approach of a survey and semi-structured interviews amongst IRs.

From the survey it was concluded that IR employees generally found that there is a need for sustainability within IR, but that action was still limited. Reasons for these limited actions were derived from semi-structured intervews and were found to be 1) lack of priority, 2) high dependency on employees and their behaviour and 3) external factors that cannot be changed, like technical and legal barriers. While implementing sustainable workflows, the convenience for employees should be guaranteed. Potential improvements could be found in a better collaboration between IR departments and a structural change from looking at sustainability as a bottom-up activity by individuals towards a more thorough approach, which includes a more proactive attitude of higher management and governmental institutions.

## Supplementary Information

**Additional file 1: Appendix**

## Data Availability

The datasets used and/or analyzed during the current study are available from the corresponding author on reasonable request.

## Abbreviations

CIRSE: Cardiovascular and Interventional Radiological Society of Europe
CT: Computed tomography
HVAC: Heating, ventilation and air conditioning
IR: Interventional radiology
LCA: Life cycle assessment
LUMC: Leiden University Medical Centre
MRI: Magnetic resonance imaging
NVIR: Nederlandse Vereniging voor InterventieRadiologie (Dutch Society of interventional radiology)
PACS: Picture Archiving and Communication System
SIR: Society of Interventional Radiology
