# Supplementary material for: Sustainability within interventional radiology: opportunities and hurdles
Source: CVIR Endovasc. 2023 Mar 20;6:16. doi: 10.1186/s42155-023-00362-1 (PMC10027964; doi:10.1186/s42155-023-00362-1)
Supplement: Supplementary file 1 — Additional file 1: Appendix [file 42155_2023_362_MOESM1_ESM.docx]

# Appendix

The following set of questions was used as a guideline for the semi-structured interviews.

1. Personal:

- Can you briefly introduce yourself and describe your job?
- What is your experience with sustainability?
- Where does your motivation come from?

2. Current state sustainability IR department:

- What is currently happening in the IR department with regards to sustainability?
- Follow-up: Energy (in detail): do you have procedures regarding shutting down equipment?
  - Scanners
  - Lights/monitors
  - HVAC/climate system
- Follow-up: Waste
  - Standard packages
  - Separating waste:
    - Knowledge of staff, education?
    - Design and position bins
    - Feedback waste separation
  - Do people know what happens with waste after separating (= recycling)?
- Follow-up: water pollution
  - Are you aware of water pollution caused by IR?
    - If yes: have you undertaken any actions?

3. Organization:

- When people have an idea, what happens with this idea? Do they know where to go?
  - Follow-up: Do you feel that people have any ideas at all, or are they just not that into it?
- What do you do in terms of sharing knowledge about sustainability with other departments (internal/external)?
- What are you running into, what are your greatest challenges?
- Follow-up: Specific challenges
  - Support base?
  - Technical difficulties?
- What do you think would be a solution (to start with)?
